# Oligothiophenes as Fluorescent Markers for Biological Applications

**DOI:** 10.3390/molecules17010910

**Published:** 2012-01-18

**Authors:** Massimo L. Capobianco, Giovanna Barbarella, Antonio Manetto

**Affiliations:** 1 Istituto per la Sintesi Organica e la Fotoreattività, Consiglio Nazionale delle Ricerche (ISOF-CNR), Via Gobetti 101, Bologna 40129, Italy; 2 ISOF-CNR & Mediteknology s.r.l., Via Gobetti 101, Bologna 40129, Italy; Email: barbarella@isof.cnr.it; 3 Baseclick GmbH, Bahnhofstrasse 9-15, Tutzing 82327, Germany; Email: a.manetto@baseclick.eu

**Keywords:** oligothiophene, fluorophore, oligonucleotide, monoclonal antibody, cell staining

## Abstract

This paper summarizes some of our results on the application of oligothiophenes as fluorescent markers for biological studies. The oligomers of thiophene, widely known for their semiconductor properties in organic electronics, are also fluorescent compounds characterized by chemical and optical stability, high absorbance and quantum yield. Their fluorescent emission can be easily modulated via organic synthesis by changing the number of thiophene rings and the nature of side-chains. This review shows how oligothiophenes can be derivatized with active groups such as phosphoramidite, *N*-hydroxysuccinimidyl and 4-sulfotetrafluorophenyl esters, isothiocyanate and azide by which the (bio)molecules of interest can be covalently bound. This paper also describes how molecules such as oligonucleotides, proteins and even nanoparticles, tagged with oligothiophenes, can be used in experiments ranging from hybridization studies to imaging of fixed and living cells. Finally, a few multilabeling experiments are described.

## 1. Introduction

Staining of biological samples has always been a powerful technique, whose milestones are represented by: the staining of neuronal tissues with silver salts developed by Golgi in 1873 [1] that allowed him to visualize the structure of neurons; the coloration of bacteria developed by Gram in 1884 [2] that allows to classify bacteria based on the physical properties of their cell walls; and the method of Giemsa that allowed him to recognize cells infected with malaria parasites [3] and is used nowadays to visualize chromosomes. In modern instruments, visible dyes are being replaced by fluorescent ones, for a plethora of biotechnological applications like FACS, DNA sequencing, FRET, and more recently by nanodots for plasmonic photothermal therapy [4] or as drug delivery agents [5].

In principle the labeling of biological molecules with visible or fluorescent dyes can alter the functioning of the colored molecules, for instance by modifying their lipophilicity or sterically interfering with receptors or substrates; also the chemistry behind the labeling process may be incompatible with the nature of the biomolecule, finally the properties of the fluorescent dyes such as emitted color, quantum yield, stability to bleaching and so on can be important for the experiment to be conducted. For all these reasons it is important to have a variety of families of fluorescent markers to count on. 

This mini-review focuses on some applications derived from our recent works on the exploitation of oligothiophenes. This class of molecules is, since long, widely studied for its use in semiconductors in devices such as field-effect transistors or solar cells [6,7], but their fluorescence properties and synthetic flexibility make them robust markers with emission in the full range of UV-Vis spectra [8]. In addition, their polarizability makes them prone to sense their microenvironment and consequently suitable to be used in recognition of biochemical process and for the realization of biosensors.

## 2. Labeling of Nucleosides and Oligonucleotides

5-(2-Hydroxyethyl)-2,2':5',2''-terthiophene was the first oligothiophene to be used as a fluorescent label for oligonucleotides [9]. The compound has an emission at λ = 441 when excited at 356 nm, it has a moderate solubility in water and its emission remains unchanged when mixed with oligonucleotides either as single or double stranded filaments, so in 2004 we decided to check its suitability for transformation into a phosphoramidite for use as a 5'-dye on a standard oligonucleotide synthesizer.

**Scheme 1:**
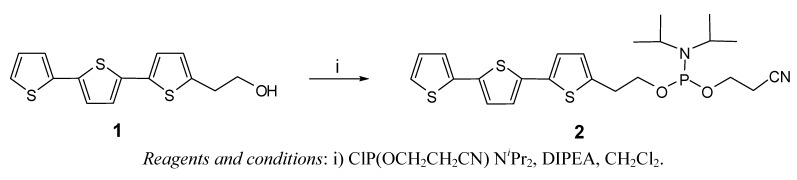
Preparation of the phosphoramidite **2**.

Terthiophene **1** was thus transformed into the phosphoramidite reagent **2** (Scheme 1) and used to prepare fluorescent oligonucleotides.

**Figure 1:**
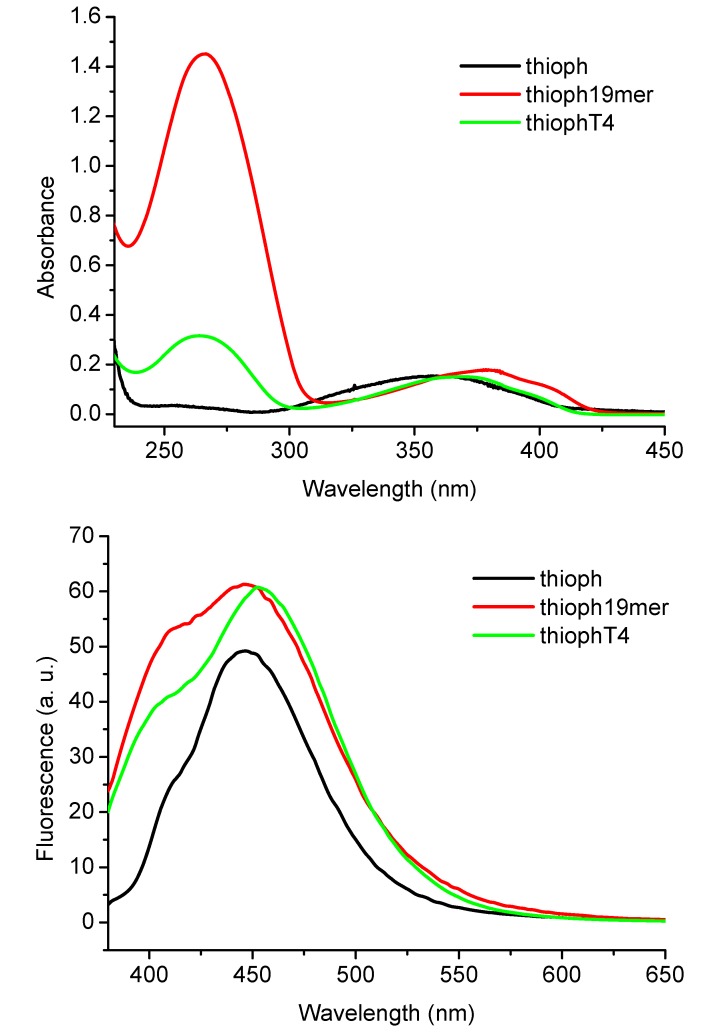
Absorbance (top panel) and fluorescence emission (bottom panel) of: oligothiopene **1** (black line), conjugates **19-mer-oligothiophene** (red line) and tetrathymidine **T_4_-oligothiophene conjugate** (green line).

It was found to react like any commercial phosphoramidite on the synthesizer and to be resistant during the hot aqueous ammonia treatment required by the solid phase cleavage and the deblocking of the protected oligonucleotides. A tetrathymidine **T_4_**, and a **19-mer** oligonucleotide were synthesized and fully characterized (by NMR and MS) and were found to maintain the fluorescence characteristics of the native oligothiophene, with no bleaching following exposition to light (Figure 1).

Following this methodology several phosphoramidites and oligonucleotides have been synthesized in our laboratories. 

A different method of labeling oligonucleotides can be that of reacting a succinimidyl derivative of the dye with an aliphatic amino group, previously attached to the oligonucleotide. We have used this route to synthesize a series of oligonucleotides to be used as molecular beacons. 

**Scheme 2:**
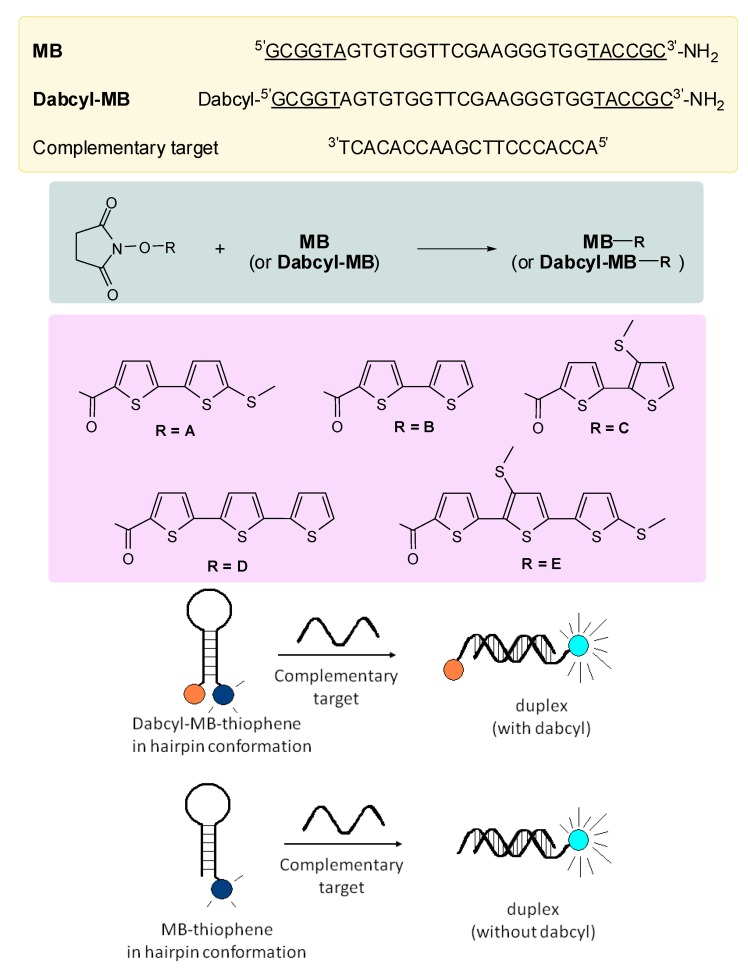
Yellow panel: oligonucleotide sequences, the underlined bases form the hairpin region. Green panel: labeling reaction. Pink panel: list of fluorophores. Cartoon: schematic representation of the hybridization of the molecular beacon (MB) with its complementary target, to give a duplex with or without the dabcyl quencher (orange circle). In successfully experiments, the emission of the fluorophore (blue circles) increases (cyan circles) passing from the hairpin configuration to the extended duplex form.

Our study model was based on the derivatization of an oligonucleotide having the possibility to assume a hairpin configuration, with a free amino group on its 3'-end, with and without, a dabcyl moiety on its 5'-end. We then prepared a series of oligothiophene succinimidyl derivatives that we used to react with the amino group of the oligonucleotide. With each thiophene derivative **A** to **E**, we prepared a couple of fluorescent oligonucleotides with and without the dabcyl moiety at the opposing end (Scheme 2).

**Figure 2:**
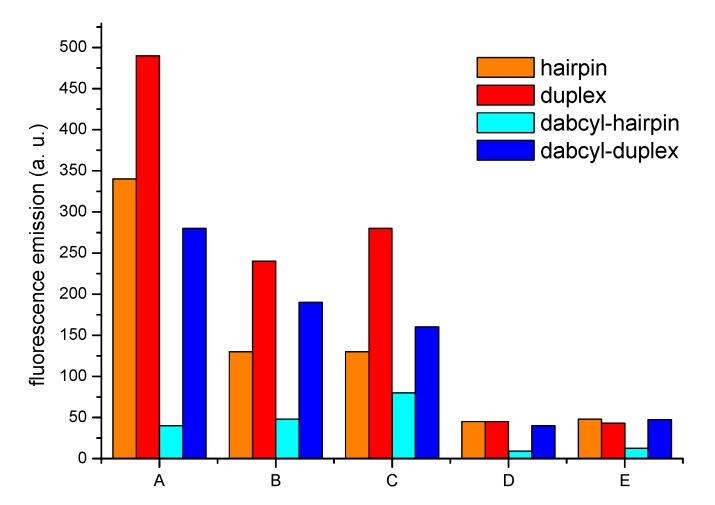
Fluorescence emission of each derivative (**A**–**E**) as: hairpin (orange), duplex (red), hairpin with dabcyl quencher (cyan), and duplex with dabcyl quencher (blue). All the experiments were performed at the same concentration of the respective oligonucleotides at room temperature.

We then studied the fluorescence behavior of each oligonucleotide (with and without dabcyl) either alone and in presence of an oligonucleotide complementary to the region of the hairpin loop, at different temperatures, to check if the oligothiophene emission was altered by the conformation and by the proximity to the dabcyl used as a fluorescence quencher, like in the right panel of Scheme 2. For each fluorophore we observed minor variations in absorption and emission wavelength (see Table 1) respect to the succinimidyl ester precursor, and more interestingly we observed a moderate to strong variation of the emitted light depending on the hybridization status in some cases even in absence of the dabcyl moiety. Our results are summarized in Figure 2.

**Table 1:** List of absorption and emission frequencies of oligothiophene N-scuccinimidyl esters and the corresponding oligonucleotide derivatives.

Derivative	Succinimidyl ester		Oligo conjugate	
	λ_ex_	λ_em_	λ_ex_	λ_em_
**A**	370	479	360	479
**B**	352	418	335	410
**C**	361	479	350	445
**D**	390	460	410	480
**E**	418	549	425	520

As indicated in the Figure 2, the dabcyl was able to quench the fluorescence of every derivative (cfr. cyan and blue bars) when the molecular beacon was folded into a hairpin conformation that forces the dabcyl to be in proximity of the fluorophore, while it cannot have such effect in the presence of the duplex that puts the extremities of the modified oligonucleotide far away. What was not anticipated, was the finding that oligothiophenes **A**, **B**, and **C** showed an important variation of fluorescence even in absence of the quencher (orange and red bars), thus indicating, in some cases the suitability of oligothiophenes “to sense” their environment in a degree that can potentially be exploited for the realization of bio-sensors even without the need of a double labeling of the oligonucleotide.

**Scheme 3:**
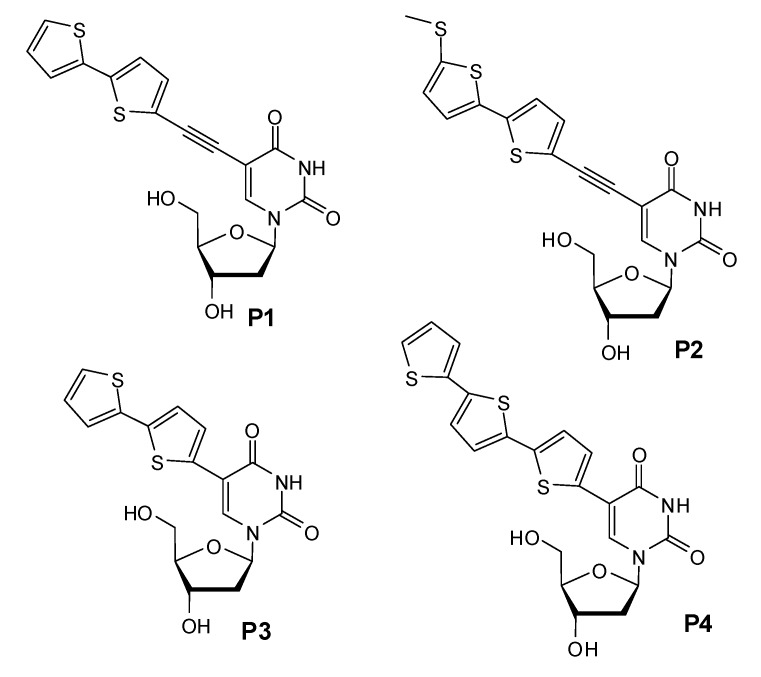
The 5-thiophene conjugated deoxyuridines used in the work.

To further explore the sensitivity of oligothiophenes to their molecular environment, we synthesized four derivatives of the deoxyuridine with oligothiophenes linked to the C5 position [10], in such a way that the aromatic rings of the oligothiophene could be coupled with the uridine base directly, or thought an ethyne spacer, so as to allow the delocalization of the base’s electrons into the thiophene rings, with the naïf idea of provoking some variation of the emitted fluorescence if the base was engaged in a Watson-Crick bond with a facing adenosine, or faced with a non complementary base. Such kind of “recognition” can indeed be used for the detection of single nucleotide polymorphism (SNP) often associated with genetic diseases. In our work we prepared the four then unknown labeled deoxyuridines shown in Scheme 3.

**Figure 3:**
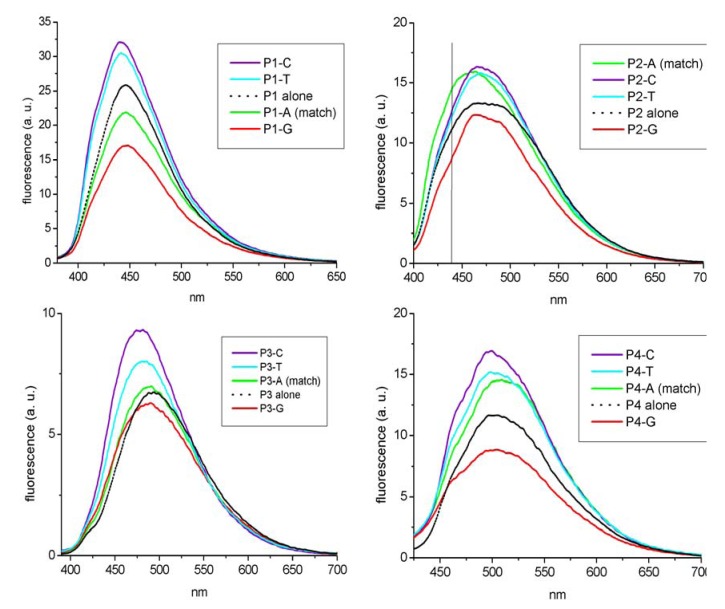
Fluorescence emission of probes **P1** to **P4** alone (black lines) or after hybridization with **targetA** (green lines), **T** (cyan lines), **C** (violet lines) and **G** (red lines). Generally there is a remarkable difference in the intensity of the emitted light between of the hybrids, depending on the base facing the modified uridines. Sometime as in the case of **P2** the differences can be better observed at a wavelength different from that of the maximum of emission f. i. at 440 nm (along the line).

We inserted them in the middle of an oligonucleotide sequence (Table 2) to be used as a probe for the detection of an SNP mutation (A to G transition) that is responsible for the production of a mutated form of hemoglobin in affected patients. The variation of the fluorescence of the probes containing the modified uridines is shown in Figure 3.

**Table 2:** Oligonucleotides used in the work.

**P1-4**	^3^' AC TGA GGA ***U***TC CTC TTC A ^5^'
**Target-A**	^5^' TG ACT CCT ***A***AG GAG AAG T ^3^'
**Target-G**	^5^' TG ACT CCT ***G***AG GAG AAG T ^3^'
**Target-C**	^5^' TG ACT CCT ***C***AG GAG AAG T ^3^'
**Target-T**	^5^' TG ACT CCT ***T***AG GAG AAG T ^3^'
***U*** = modified deoxyuridine (compounds **1–4** as in Scheme 3)	

As the panels of Figure 3 show, the emitted fluorescence of the probes alone is intermediate between those of the correctly hybridized probe and that of the probes facing the G mismatch. In some cases also the mismatches with C and T can be recognized. This behavior could be exploited in diagnostic tests and sensors.

A more recent methodology for labeling biomolecules is the one that uses the reaction between the azido group and a terminal alkynyl group to form a triazolyl-conjugate between the molecules carrying the above mentioned moieties [11]. This reaction is an example of the click-chemistry concept theorized by Sharpless in 2001 [12], improved by the use of catalysts and ligands, such as Cu(I) ions and the tertiary amine tris-(benzyltriazolylmethyl)amine (TBTA) [13] respectively and widely known as Copper(I)-catalyzed Azide-Alkyne Cycloaddition (CuAAC). The CuAAC reaction, which was introduced independently by Meldal [14] and Sharpless [15] in 2002, occurs smoothly and quantitatively, even in aqueous solutions and at room temperature, with a predictable 1–4 regiochemistry. Remarkably, the CuAAC reaction is highly bioorthogonal, as neither azide nor terminal alkyne functional groups are generally present in natural systems placing the CuAAC reaction in an excellent position to take over as the state-of-the-art methodology to label and modify DNA and other biomolecules.

Several examples of oligothiophene-oligonucleotides have been prepared by our groups (ISOF and baseclick) using the CuAAC reaction with astonishing results in term of obtained labeling yields, emitted colors and quantum yields.

**Scheme 4:**
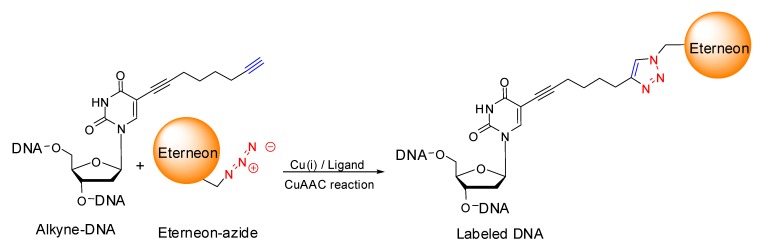
Click chemistry principle applied to DNA labeling. The oligothiophene azide (Eterneon^TM^-N3) is post-synthetically introduced in the oligonucleotide via the CuAAC reaction.

We used a post-synthetic approach to introduce oligothiophenes in oligonucleotides, firstly synthesizing alkyne-containing oligonucleotides via solid phase synthesis and secondly labeling them via CuAAC reaction using a small excess–2 equivalents–of the oligothiophene azido-derivates, reported herein with their commercial name “Eterneon^TM^ azides”. After the addition of pre-complexed Cu(I)/ligand, complete conversion to the labeled oligonucleotide is observed in a time span between 30 min and 4 h. Following a simple precipitation step, the labeled oligonucleotides can be recovered in near quantitative yields (Scheme 4).

To demonstrate the usefulness of oligothiophene-azides as fluorescent markers for oligonucleotides with the CuAAC reaction, we prepared several derivatives starting with the following oligonucleotides:

**16-mer**: sequence: ^5'^GCG CTG T**X**C ATT CGC G^3'^
**22-mer**: sequence: ^5'^**X**CG AT**X** GCA T**X**A GCC A**X**T AT**X** C^3'^
**38-mer**: sequence: ^5'^**X**TT A**X**T GT**X** TTA **X**GC C**X**A TT**X** TTT **X**AT G**X**T TT**X** AGC **X**T^3'^

where **X** is a modified deoxythimidine with a C_8_ alkyne attached to the C5 position of the base, as depicted in Scheme 4.

The modified oligonucleotides were synthesized via solid phase synthesis, using standard protocols and standard phosphoramidites along with the C8-alkyne-dT-phosphoramidite (baseclick GmbH) as shown in the Scheme 4 as part of the oligonucleotide named “Alkyne-DNA”. The incorporated internal alkyne of the **16-mer** was reacted with two equivalents of Eterneon^TM^-(480/635)-azide for 3 h at 37 °C in presence of a Cu(I)/TBTA pre-complexed mixture (baseclick GmbH). 98% of the labeled oligonucleotide was recovered from the following ethanol precipitation.

**Figure 4:**
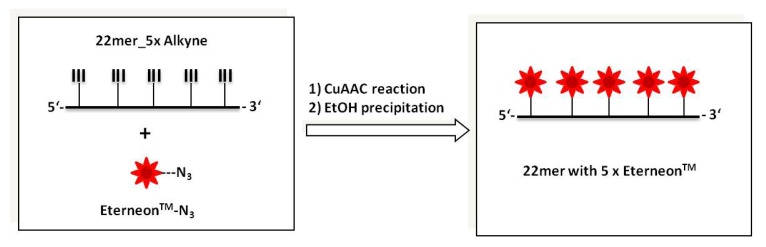
Graphic representation of high density functionalization via click chemistry (CuAAC reaction) of oligonucleotides with oligothiophene-azide (Eterneon^TM^-azide).

The high efficacy of the CuAAC reaction enables the multiple post synthetic oligothiophene labeling of alkyne modified nucleic acids as well. Complete high-density functionalization of several alkyne moieties within the oligonucleotides can be achieved without the formation of by-products as shown in the graphical representation (Figure 4) and reported in the examples below.

**Figure 5:**
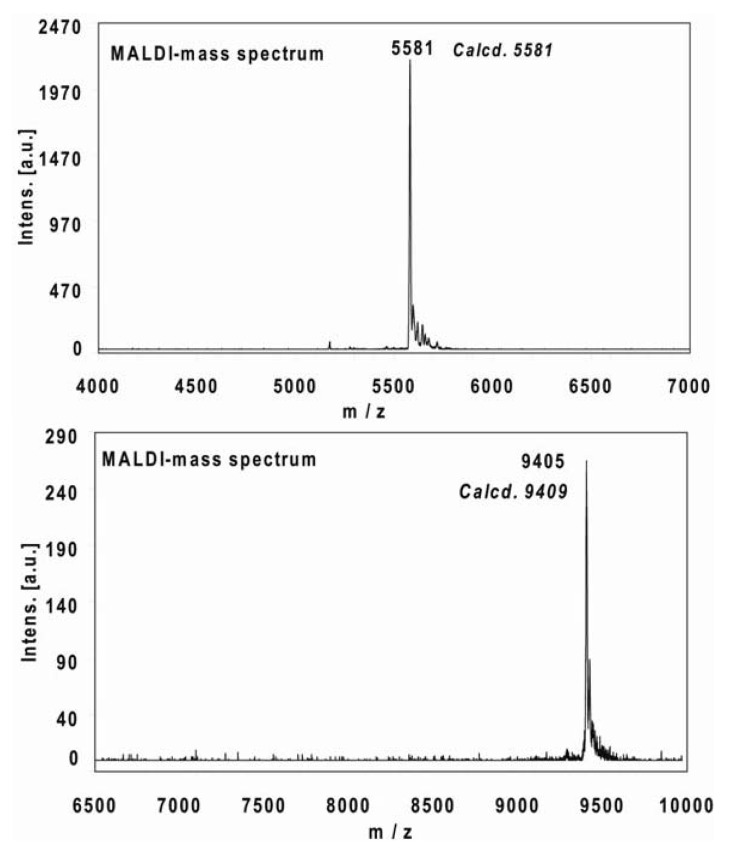
MALDI-mass spectrum of the crude conjugation reaction of **16-mer** oligonucleotide internally labeled with the oligothiophene Eterneon^TM^-480/635 azide, after the precipitation step without further purification (first panel). MALDI-mass spectrum of the crude conjugation reaction of the **22-mer** with the oligothiophene Eterneon^TM^ 350/430 azide after the precipitation step without further purification (second panel) (unpublished data).

Using the above described procedure, the five-fold and ten-fold derivatives of the modified oligonucleotides **22-me**r and **38-mer** were obtained. The compounds identification was assessed by Maldi-mass analysis, of which some examples are reported in Figure 5.

**Figure 6:**
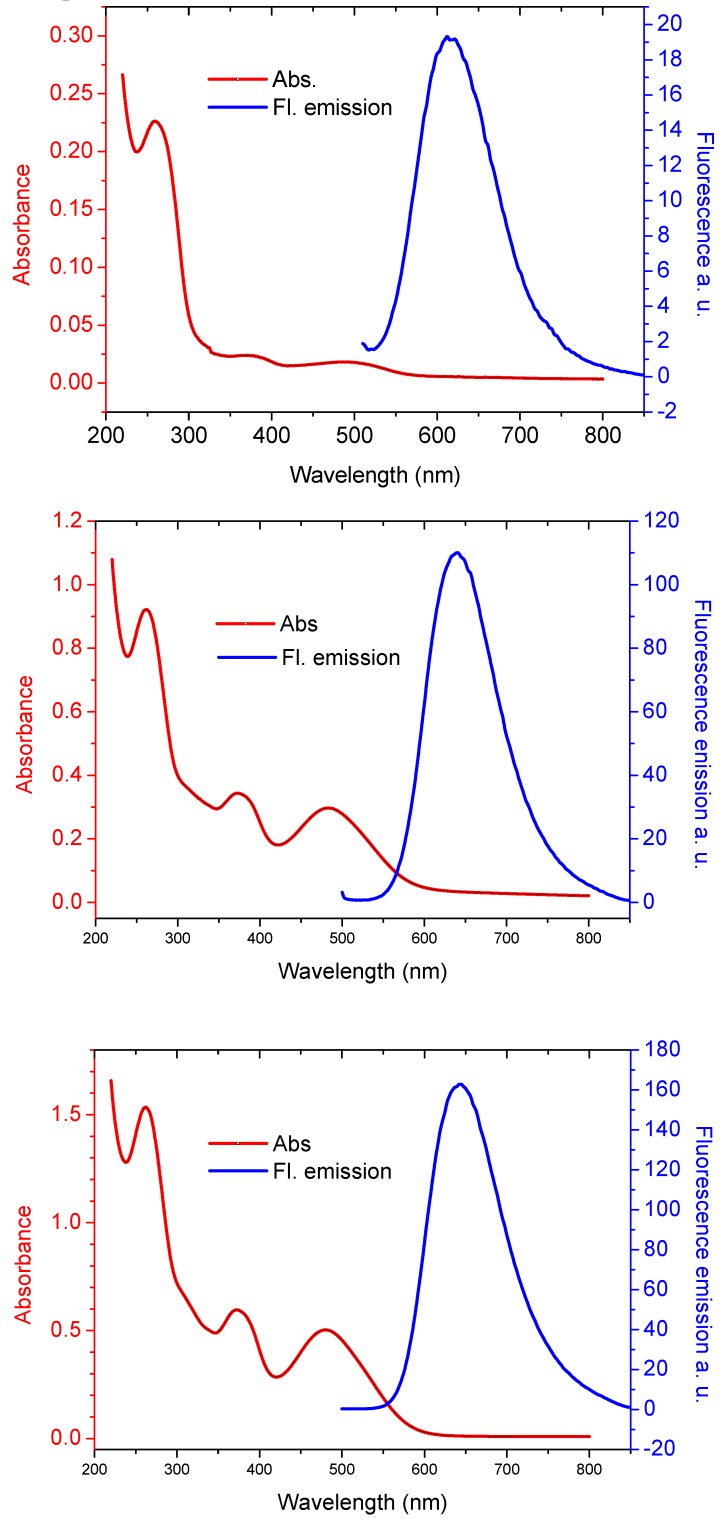
UV absorption (red line) and fluorescence emission (blue line) of oligonucleotides conjugated with Eterneon^TM^-480/635 azide. **16-mer** monolabeled conjugate at 1.61 μM concentration (first panel), **22-mer** five times labeled conjugate at 4.46 μM concentration (second panel) and **38-mer** ten times labeled conjugate at 4.47 μM concentration (third panel) (unpublished data).

The comparison of the mono and the multiple labeled (5-fold and 10-fold) derivatives of the oligonucleotides shows that the effect of the multiple labeling is not purely additive (Figure 6), as demonstrated by the intensity of the emitted fluorescence and by its color (position of the maximum), but a discussion of these phenomena is beyond the scope of this review and will be reported in a subsequent paper.

**Figure 7:**
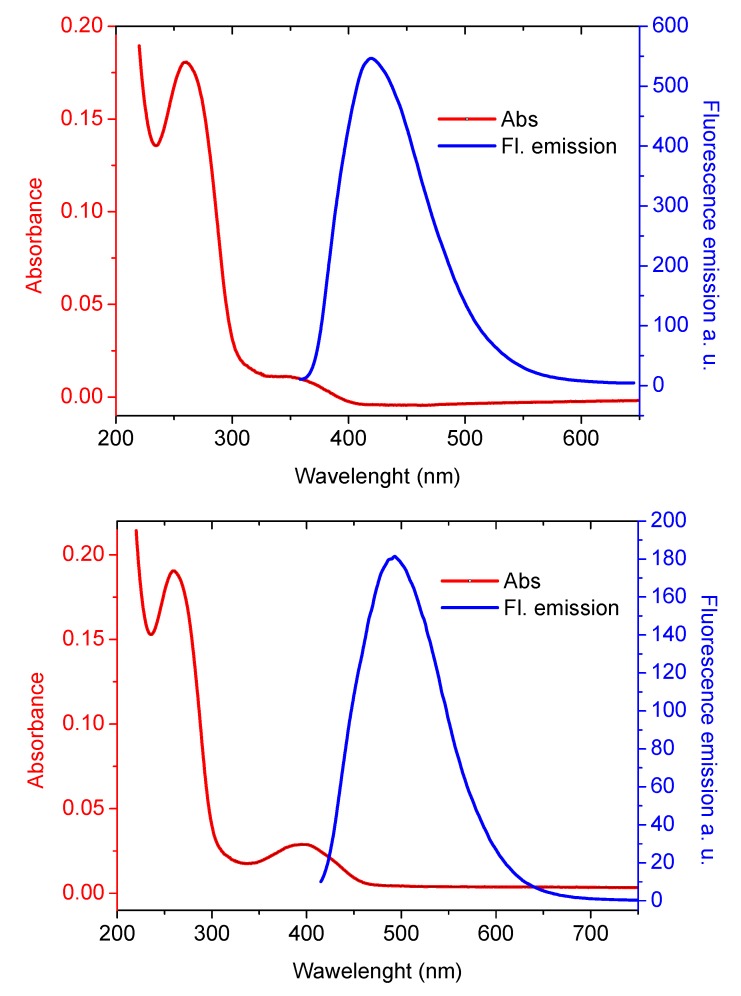
UV absorption (red line) and fluorescence emission (blue line) of **16-mer** conjugated with Eterneon^TM^ 350/430 azide at 1.29 μM (top panel); and **16-mer** labeled with Eterneon^TM^ 348/480 azide (bottom panel) at 1.35 μM concentration (unpublished data).

Several oligothiophene derivatives that differ for the number of rings and the presence and positioning of substituents are available for this methodology, each one characterized by a different color and emission intensity. Two of them are shown in Figure 7.

This click chemistry approach as labeling method offers additional advantages regarding the stability of reagents, intermediates and final products as well. Both click chemistry partners are in fact very stable even in aqueous solutions, allowing long storage times and easy handling without affecting the efficacy of the reaction. Thus the oligothiophenes “Eterneon^TM^ azides” offer both the photostability typical of this class of fluorophores, which is higher than the common fluorescent dyes, and a long shelf-life typical for the azido reactive group, much longer than the shelf-life reported for the succinimidyl derivates used in the standard amino-reactive methods.

Further applications involving the oligothiophene labeling of nucleic acids are under investigations. Fluorescent *In Situ* Hybridization (FISH) experiments, for example, may take enormous advantage by the use of multi-labeled probes in which the fluorophores show high photostability, allowing long exposition times and higher signal to noise ratio.

## 3. Labeling of Proteins and Antibodies

One remarkable characteristic of oligothiophenes as fluorophores is that several emission shades can be designed and realized by an easily planned synthesis of monomers and simple substituents. This allows one to work with a single family of dyes, therefore making easier the task of scientists that can expect for each derivative the same chemical behavior in term of photostability, sharp spectral emission, high absorbance, high fluorescence quantum yield, large differences between absorption and emission wavelengths (Stokes shifts), color tunability from blue to red to white, easy binding to biomolecules, lack of toxicity, and low preparation costs. All these advantages can be found in the still growing number of derivatives synthesized at the ISOF and Mediteknology laboratories.

**Scheme 5:**
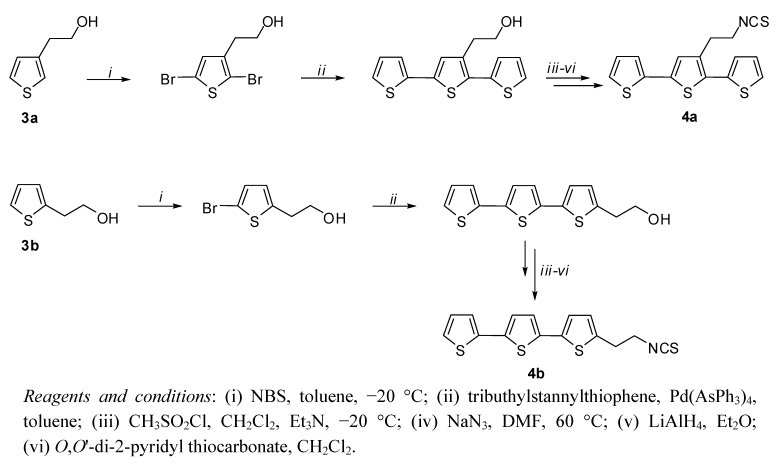
Schematic representation of the synthesis of isothiocyanates **4a** and **4b** starting from the hydroxyethyl derivatives of thiophene **3a** and **3b**.

The first approach toward fluorescent markers able to react with the amino-groups of biomolecules was accomplished in 2001 with the synthesis of isothiocyanate derivatives [16] of terthiophene (Scheme 5). Those molecules resulting from the following Scheme: alcohol, mesylate, azide, amine, isothiocyanate, were found to be able to be conjugated with bovine serum albumin (BSA) and anti-CD-8 monoclonal antibody with high efficiency, leading to conjugates with high and persistent fluorescence. 

**Figure 8:**
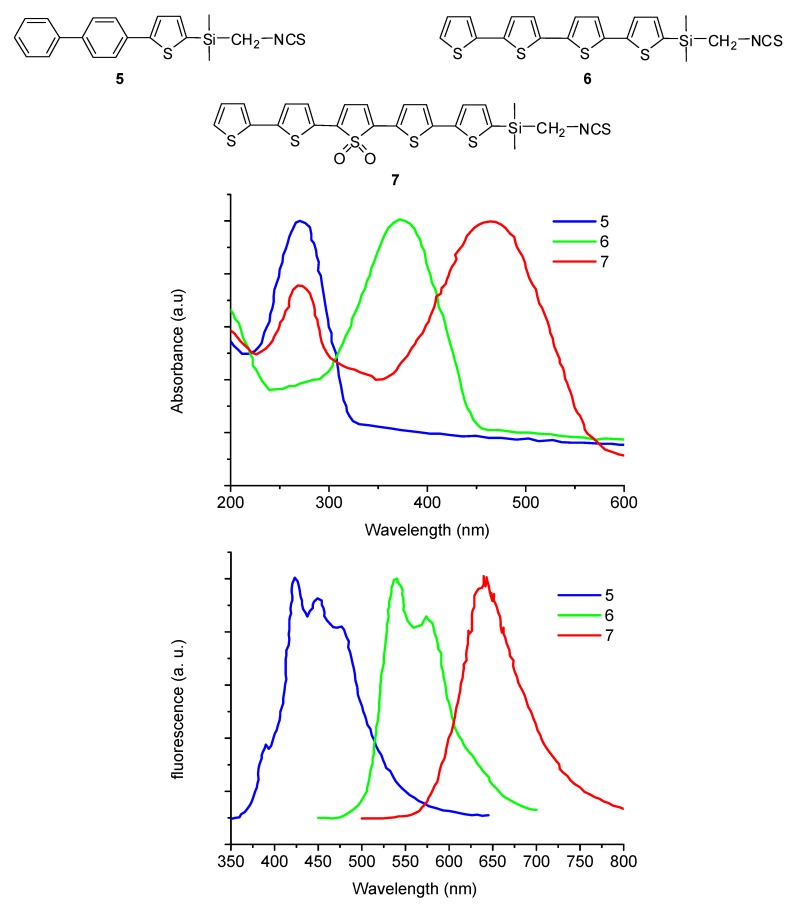
Structure of isothiocyanates **5**–**7** and their UV absorbance (top) and fluorescence emission (bottom) spectra.

Compound **4b** was used to label bovine serum albumin (BSA) and it was found that up to 30 molecules of the oligothiophene could be bound to the BSA through the ε amino group of some of the 59 lysine residues present, before affecting the solubility of the protein in a saline buffer solution, an evidence of non-denaturation. Compounds **5**–**7** were similarly obtained. They were found to fluoresce in the range from 350 to 750 nm with very little overlapping, thus allowing multicolor experiment to be performed simultaneously (Figure 8).

In particular compound **7** was used to label anti-CD8-monoclonal antibodies that could be detected which a commercial fluorescence microscope.

**Scheme 6:**
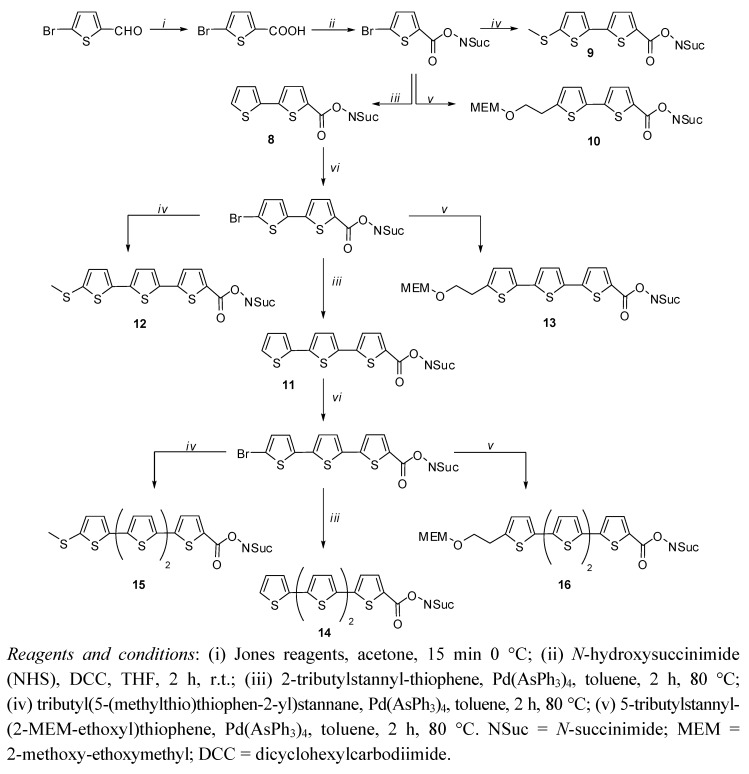
Synthesis of NHS esters.

A different way of conjugation with the amino-residues of biomolecules is the one that uses the *N*-hydroxysuccinimidyl esters (NHS) (Scheme 6) whose preparations were reported in a 2006 paper [17]. In that paper it is shown how a large number of derivatives can be prepared by repeated reactions of bromination, oxidation and Stille coupling, followed by a careful chromatographic purification.

**Figure 9:**
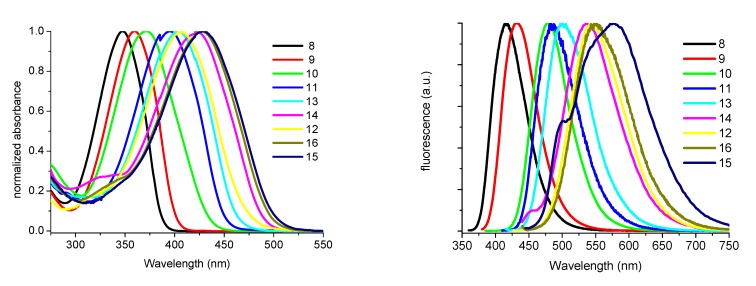
Normalized absorption (left) and normalized fluorescence emission (right) spectra of compounds **8**–**16** in CH_2_Cl_2_.

As mentioned above, the synthesized fluorophores cover the full visible range of emitting colors and their sharp spectra can be used for multicolor experiments (Figure 9). Oligothiophene succinimidyl esters can be reacted with the ε-NH_2_ residues of Lys group of proteins to make stable amide bond with the oligothiophene residue.

**Scheme 7:**
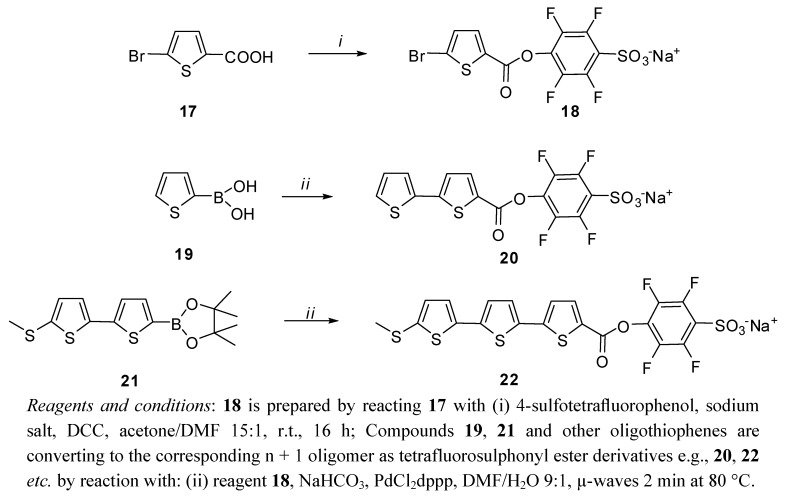
Synthesis of tetrafluorophenylsulphonyl esters of oligothiophenes.

Alternative reacting oligothiophene agents are the sulphonyl-tetrafluorophenyl (STP) esters. These reactive esters can be conveniently made starting from the commercial 5-bromo-2-thiophenecarboxylic acid (by reacting it with 4-sulpho-tetrafluorophenol and DCC), then incorporating this ring into longer oligomers obtained by sequential Pd(II) Suzuki cross-coupling reactions on halogenated and boronated building block (Scheme 7).

The final incorporation of the fluorinated active ester can be achieved reacting the appropriate boronic precursors with 18 for two minutes using microwave irradiation, and avoiding aqueous workup. This simple protocol is cost effective and can be scaled up to 300 mg at a time, leading to molecules that are still more reactive than the *N*-hydroxysuccinimidyl analogs [18] and can be used to label (bio)molecules bearing free amino groups.

## 4. Cell Staining

Oligomers of thiophene are generally non toxic [19] and this makes them particularly suitable to be used for cellular staining and observation with fluorescence microscopy.

Fluorescent thiophene succinimidyl esters are bright enough for effective cell labeling and are characterized by high photostability, so that their characteristics are not appreciably modified under the high excitation intensity at the focus of the objective of a fluorescence microscope. Moreover, labeled cells observed after several days show no sign of decline of the optical characteristics. So far we have carried out several kinds of cellular experiments in which cells have been made fluorescent after binding with oligothiophene fluorophores in both specific (binding of antibodies) and non-specific way (uptake of doped nanoparticles). 

### 4.1. Staining of Fixed Cells with Monoclonal Antibodies Labeled with Thiophene Fluorophores

A few examples of fixed cells staining with thiophene fluorophores emitting in different colors are shown below. Other examples can be found in references [17,18].

**Figure 10:**
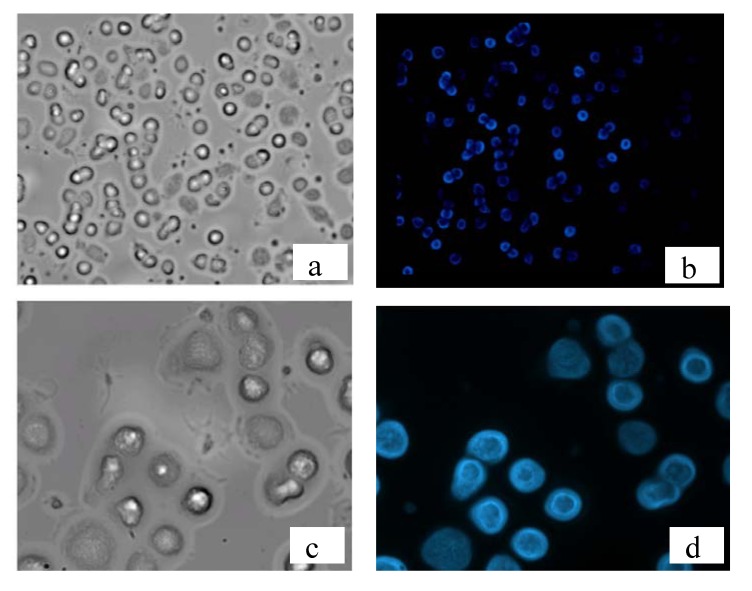
T lymphocytes from a fresh blood sample of a healthy volunteer stained using anti-CD3 mAb labeled with Eterneon^TM^ 350/455 NHS Light transmission (**a**, **c**) and fluorescence (**b**, **d**) images. Top: magnification 40×. Bottom: magnification 100×.

Figure 10 shows the optical and fluorescence microscopy images of T lymphocytes of a healthy volunteer stained using the anti-CD3 monoclonal antibody labeled with a cyan fluorescent thiophene dye. Anti-CD3 (Clone BB12) is a Mouse IgG1 isotype monoclonal antibody reacting with CD3, a 20 kDa molecular weight antigen normally expressed on the surface of T-lymphocyte cells. It binds specifically to the antigen on cell surface, causing the fluorescent emission of the membrane of the cells where the antigen is present. 

In the images of Figure 10 the staining of the cell membrane is clearly visible, as well as the distinction between labeled and unlabeled cells in comparison with the optical microscopy image. Remarkably, the bright cyan fluorescence was still evident on cells membrane of the same sample two months after the first examination.

**Figure 11:**
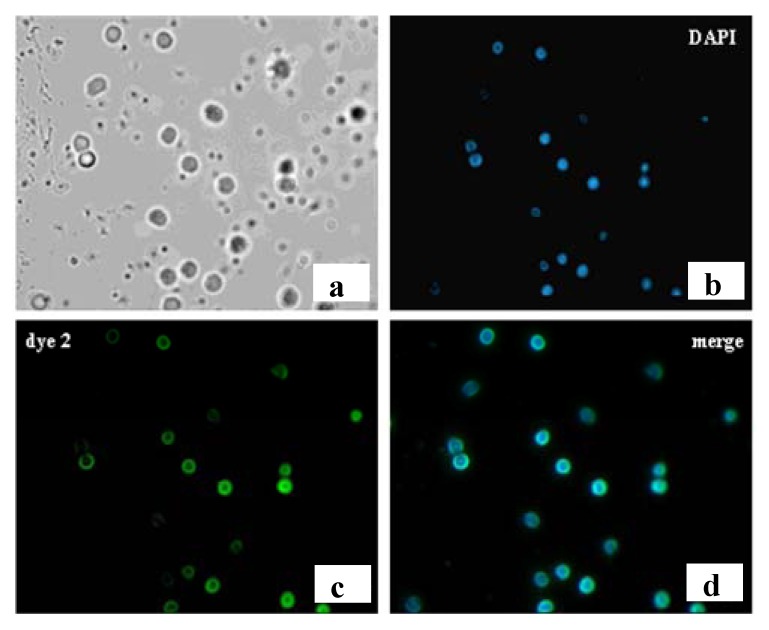
T lymphocytes from a fresh blood sample of a healthy volunteer stained using anti-CD4 mAb labeled with Eterneon^TM^ 384/480 NHS. (**a**) Light transmission image; (**b**) Nuclear staining with DAPI; (**c**) Fluorescent localization of the anti-CD4 labeled with dye 2 showing that the fluorophore is localized exclusively in the membrane of approximately the 50% of the cells, without entering in the cytosol; (**d**) Merge image (**b** + **c**) showing nuclear and membrane staining. Magnification: 40×.

Figure 11c shows the green fluorescent staining of the membrane of T-lymphocytes with the conjugate of the anti-CD4 monoclonal antibody with a green emitting thiophene fluorophore. The Figure 11 also shows (panel b) the counterstaining of the cells with DAPI, that colors the nuclei of both labeled and unlabeled cells, and the corresponding merging image (panel d). DAPI is a blue emitting dye which colors selectively the cell nucleus. The figure shows that there is good fluorescence discrimination between labeled and unlabeled cells. 

**Figure 12:**
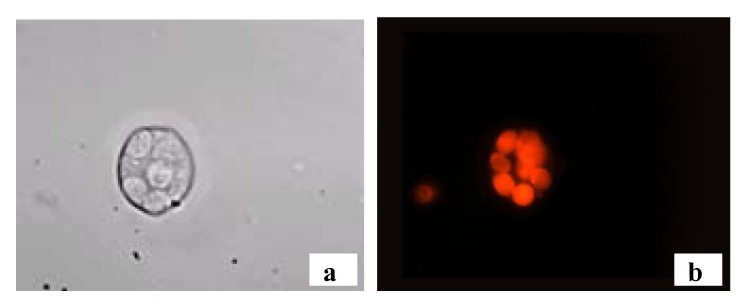
T lymphocytes from a fresh blood sample of a healthy volunteer stained using anti-CD3 mAb labeled with **Eterneon^TM^** **394/507NHS**. Light transmission (**a**) and fluorescence (**b**) microscopy images, magnification: 100×.

Figure 12 illustrates the staining of T lymphocytes with the anti-CD3 conjugated with an orange-red emitting thiophene fluorophore. A very bright orange-red fluorescence of the membrane of the cells can be observed [8].

### 4.2. Cells Stained with Nanoparticicles Labeled with Thiophene Fluorophores

**Scheme 8:**
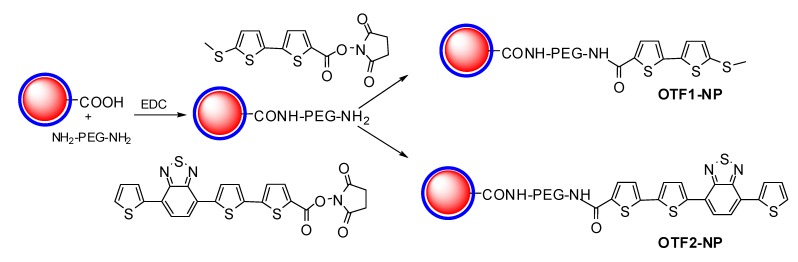
Synthesis of thiophene labeled nanoparticles.

Cells can be made fluorescent by the absorption of inorganic nanoparticles (NP) made of gold or iron oxide, conjugated with oligothiophenes as indicated in Scheme 8 [20].

**Figure 13:**
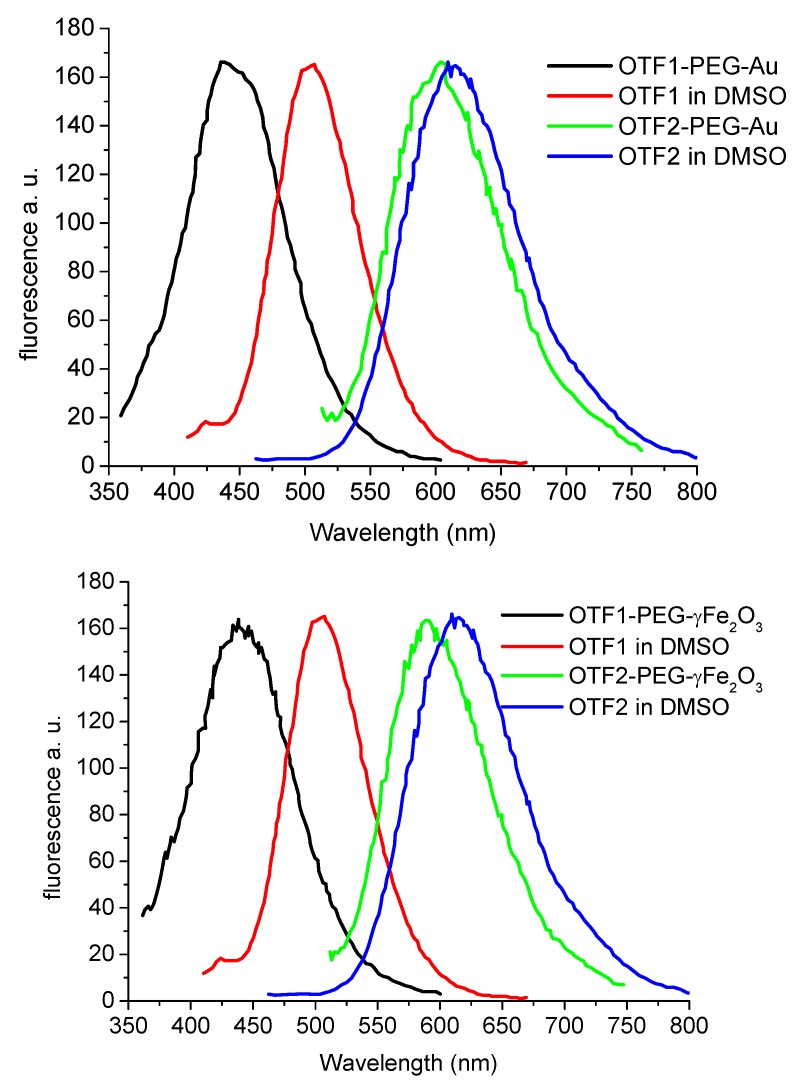
Comparison of the photoluminescence spectra of thiophene fluorophores OTF1 and OTF2 in DMSO with those of the corresponding OTF-NP conjugates. **Top panel: **OTF1-PEG897-Au and OTF2-PEG897-Au compared with nonconjugated fluorophores OTF1 and OTF2 in DMSO. **Bottom panel:** OTF1-PEG897-γ-Fe_2_O_3_ and OTF2-PEG897-γ-Fe_2_O_3_ compared with nonconjugated OTF1 and OTF2 in DMSO.

The nanoparticles (red spheres) were made water soluble wrapping them in an amphiphilic polymer shell, based on cross-linked poly anhydride molecules (the blue shell). Hydrolysis of the poly anhydride provides carboxylic groups at the surface of polymer-coated NPs, which were reacted with diamino-PEG molecules by means of the coupling agent EDC. Then the diamino-PEG NPs were reacted with the *N*-hydroxysuccinimidyl ester group of the OTFs to yield the ﬁnal conjugates by means of an amide bond formation using different OTF1 and OTF2 molecules. A comparison of their emission spectra is reported in Figure 13.

OTF-NP conjugates are chemically stable and can be stored for several months without compromising their ﬂuorescence and magnetic properties.

**Figure 14:**
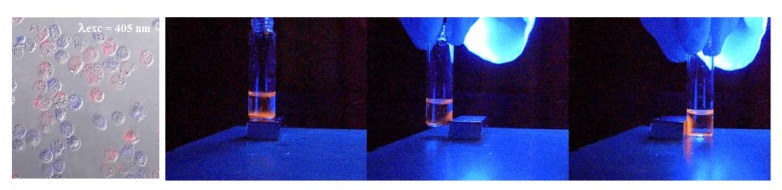
Confocal image of KB cells treated with OTF1-γ-Fe_2_O_3_ nanoparticles (blue emitting cells) and OTF2-γ-Fe_2_O_3_ nanoparticles (red emitting cells) obtained using a single excitation laser source at 405 nm (left image). KB cells treated with OTF2-γ-Fe_2_O_3_ attracted by a small magnet placed close to the vial under UV light exposure (photographs).

Magnetic fluorescent oligothiophene conjugates with iron oxide were used in cellular experiments. It was shown that they are taken up from epidermal carcinoma (KB) cells with a good (>80%) viability. The doped cells could be clearly observed under fluorescence microscopy even in multiplexing experiments with a single excitation source, namely a 405 nm excitation laser (Figure 14) [21]. Figure 14 also shows that KB cells treated with OTF2-γ-Fe_2_O_3_ put on a vial close to a small magnet move to the region near the magnet. 

### 4.3. Intelligent Surfaces

**Figure 15:**
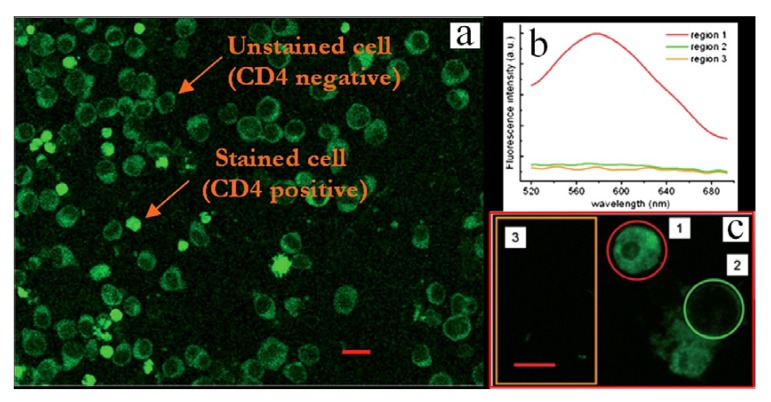
(**a**) Confocal images of Jurkat cells after the release of the anti-CD4 monoclonal antibody labeled with a thiophene fluorophore from PMMA microgels covalently attached on the surface; (**b**) PL spectra of the selected regions 1–3, (enlarged in **c**) showing that an intense PL band is only present in the correspondence of the cell (region 1). Scale bars: 20 μm for **a** and 10 μm for **c** (Reproduced by permission of The Royal Society of Chemistry, from [23]).

Recently oligothiophene-labeled BSA and monoclonal antibodies have been used to stain live cells through the use of immobilized microgels. Poly(metacrylic acid) (PMAA) microgels were immobilized on a glass surface, then loaded with the labeled proteins. This combination is in effect a “smart surface” that can be used to entrap and store the biomolecules. Allowing the competent cell to grow on those smart surfaces for some hours, they became fluorescent, absorbing the labeled proteins released by the immobilized microgel whose mesh structure can be loosened by an increased pH [22,23]. Figure 15 shows the micrograph of a suspension of Jukart cells grown at pH 7.8 on glass surface covered with PMAA previously charged with anti-CD-4 monoclonal antibodies labeled with an oligothiophene.

This strategy enables the preservation of folded (active) antibody conformations, preventing surface induced denaturation, while the immobilized microgels ensure the antibody to remain hydrated during storage, providing a signiﬁcant tool to realize fully integrated sample preparation in single-cell analysis chip and ﬂow cytometry.

A careful analysis of the distribution of the fluorescence of oligothiophene conjugated anti CD8 or CD4 monoclonal antibodies inside the PMAA gel also showed that while the former is located in the core of the microgel, the latter is mainly located in the shell part [24]. This kind of distribution might find some application in future developments of the releasing methodology. The same paper demonstrates that the treatment of Jukart cells up to 96 h at 1 or 10 mg/mL of microgels did not affect their viability.

## 5. Conclusions

Oligothiophenes are a class of molecules characterized by intense and stable fluorescence tunable over the entire visible range. This property can be tailored combining small variations in the number of thiophene rings and the presence and positioning of simple ring substituents. Their fluorescence does not bleach even under prolonged irradiation, and the oligothiophenes were found to be well tolerated even by living cells. Their transformation into isothiocyanates, *N*-hydroxy-succinimidyl, tetrafluoro-4-sulfonic-phenol esters and phosphoramidites or azide make them suitable for binding biomolecules such as proteins or nucleic acids and even nanoparticles and surfaces.

**Scheme 9:**
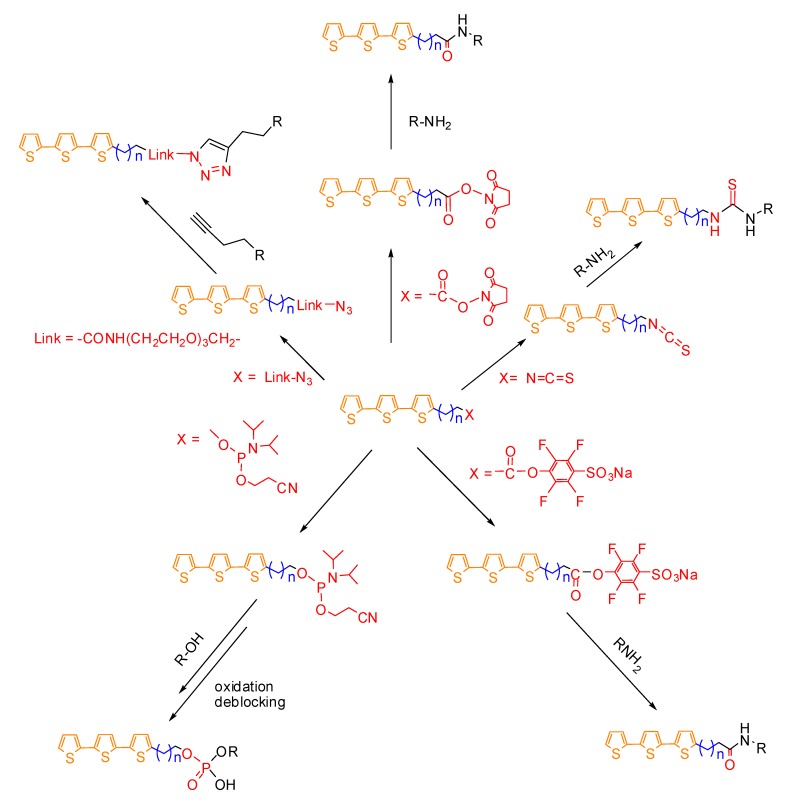
Schematic representation of the synthesis of reactive oligothiophenes (generally represented by a terthiophene) and successive labeling of biomolecules (oligonucleotides or proteins R) having free hydroxyl, amino or alkynyl groups.

Scheme 9 summarizes the different types of reactive groups used so far to conjugate oligothiophenes to biomolecules, using terttiophene as model oligothiophene. The oligothiophene-labeled biomolecules and nanoparticles can be taken up by cells in a specific or unspecific manner and made possible even the realization of multiple color experiment using a single excitation source. The oligothiophenes fluorescence, at least for some derivatives, has shown to be sensitive to the microenvironment and its variations can be used to sense biochemical processes. For all these reasons oligothiophene fluorophores represent an excellent alternative to conventional dyes.
